# Feasibility and Acceptability of a Mobile App to Improve Quality of Life of Long-Term Breast Cancer Survivors: Single-Arm Pre-Post Intervention Pilot Study

**DOI:** 10.2196/76719

**Published:** 2025-11-04

**Authors:** Nelia Soto-Ruiz, Gustavo Adolfo Pimentel Parra, Paula Escalada-Hernández, Cristina García-Vivar

**Affiliations:** 1 Department of Health Sciences, Public University of Navarre, Avda. Barañain s/n, Pamplona, 31600, Spain, (+34) 948 16 61 50; 2 Navarre Institute of Health Research, Pamplona, Spain

**Keywords:** mobile health, breast cancer survivors, quality of life, self-efficacy, feasibility study, digital health, mHealth apps

## Abstract

**Background:**

Long-term breast cancer survivors often continue to experience physical and psychological sequelae, despite being cancer-free; these challenges can negatively impact their quality of life and self-efficacy. Mobile health interventions constitute a promising strategy for providing personalized support. However, the feasibility and acceptability of these tools in long-term breast cancer survivors have not yet been sufficiently explored.

**Objective:**

This study aimed to evaluate the feasibility and acceptability of the CUMACA-M, a digital health app designed to improve the quality of life and self-efficacy in long-term breast cancer survivors.

**Methods:**

A single-arm feasibility pilot study was conducted with pre- and post-intervention evaluations. Participants were recruited from the Navarra Breast Cancer Association (Saray), a nonprofit organization supporting individuals with breast cancer in Navarra, Spain. The inclusion criteria included being female, being aged ≥18 years, having been diagnosed with breast cancer, and being disease-free for at least 5 years after primary treatment. The participants used the CUMACA-M app for 3 months. Feasibility was assessed through recruitment and completion rates, whereas acceptability was measured using the System Usability Scale and open-ended qualitative questions. Changes in quality of life and self-efficacy were analyzed with the Quality of Life-Cancer Survivors (QOL-CS) scale and the Self-Efficacy to Manage Chronic Disease Scale. Paired *t* tests were performed for pre-post comparisons.

**Results:**

A total of 23 women (mean age =52.8, SD 6.1 years) participated, with a 100% retention rate. The System Usability Scale score (mean 80.8, SD 15.2) indicated excellent usability. The health advice module received the highest level of satisfaction, whereas the nutrition and physical activity modules received suggestions for improvement. With respect to the clinical outcomes, no statistically significant differences were found between the pre- and post-intervention scores on the QOL-CS (total score: pre=5.96, SD 1.08; post=5.72, SD 1.20; *P*=.07) or the Self-Efficacy to Manage Chronic Disease Scale (total score: pre=6.57, SD 1.90; post=6.26, SD 1.82; *P*=.40). However, a reduction was observed in the QOL-CS spiritual well-being subscale (pre=5.35, SD 1.13; post=4.93, SD 1.22; *P*=.05).

**Conclusions:**

As a pioneering digital intervention for long-term breast cancer survivors, CUMACA-M appears to be a potentially viable and acceptable intervention for this population, as suggested by the high level of usability and absence of dropouts. However, the findings should be interpreted with caution because of the limited sample size and the short follow-up period. The lack of significant changes in quality of life or self-efficacy may be influenced by these constraints. Future studies with larger, more diverse samples and longer follow-up periods are needed to more robustly assess the long-term impact of this intervention.

## Introduction

Breast cancer is the second most diagnosed cancer worldwide, with 2,296,340 new cases in 2022, surpassed only by lung cancer [1]. In the same year, breast cancer caused 666,103 deaths, making it the fourth leading cause of cancer death worldwide [1].

Despite the high incidence, advances in early detection, surgical techniques, and personalized treatments have contributed to reducing mortality from breast cancer and reaching net survival rates close to 90% at 5 years and 80% at 10 years [2-5]. However, despite advances in treatments and increased long-term survival, breast cancer survivors often continue to express needs that are not covered by health services [6,7]. In many cases, treatments lead to persistent side effects and late effects, such as lymphedema, cardiotoxicity, cognitive impairment, pain, and fatigue, which affect survivors’ quality of life (QOL) [8-10]. Moreover, emotional problems such as depression, anxiety, and fear of recurrence are common [10-12]. In this context, the digital transformation of health care is gaining increasing relevance because the use of information and communication technologies in the sector allows new forms of access and monitoring for patients with complex needs [13].

The COVID-19 pandemic has accelerated these new realities in access to health services, such as telehealth and telemedicine, from any point and at any distance [13]. Thus, the use of information and communication technologies in health or digital health technologies (eHealth) is proposed as an innovative strategy for improving aspects, such as barriers due to distance, time, and cost, in addition to being a great advantage for allowing access to many patients at the same time and at any time and place [13,14].

Within the field of eHealth, mobile health—which refers to the use of mobile devices, such as smartphones, tablets, smartwatches, and other wireless devices, to support medical and public health practices—is notable [15]. These devices offer multiple functionalities, including telephone calls, sending and playing multimedia content, access to the internet, and support for computer apps, making it easier for patients to self-manage their disease [15].

Numerous digital health interventions have been implemented [16]. However, the evidence of the benefits of these interventions in cancer survivors remains limited [17], with some exceptions of studies that have demonstrated its effectiveness in improving psychological aspects, QOL, and self-efficacy [16-18]. At the international level, successful experiences with personalized digital interventions have been documented. For example, in the Netherlands, a program that provided psychosocial support and promoted healthy lifestyles among patients who had completed primary treatments (eg, surgery, chemotherapy, or radiotherapy) between 1 and 12 months after diagnosis was developed [19]. However, despite advances in this field, mobile health tools designed specifically for breast cancer survivors remain narrow in scope, as evidenced by a systematic review highlighting the scarcity of mobile apps tailored to individual needs [20]. In addition, studies focusing on the population of long-term cancer survivors, that is, people who are free of the disease for at least 5 years from the end of their treatment, are still scarce [21,22].

This study aimed to assess the feasibility and acceptability of the mobile app CUMACA-M (acronym for Care Beyond Breast Cancer, in Spanish), a digital intervention based on artificial intelligence algorithms, to improve the QOL and self-efficacy of long-term breast cancer survivors.

## Methods

### Overview

This study is a part of a larger clinical trial [23] that uses the methodological framework of the Medical Research Council for the evaluation of complex interventions [24,25]. The Medical Research Council framework consists of four phases: development (phase I), feasibility and piloting (phase II), implementation (phase III), and evaluation (phase IV). This research will focus on phase II or exploratory trials to evaluate the acceptability and feasibility of the CUMACA-M intervention.

### Design

This feasibility study involved a single-arm pilot study with pre- and post-interventions [26]. The study protocol followed the Transparent Reporting of Evaluations with Nonrandomized Designs (TREND) guidelines and was registered at ClinicalTrial.gov on April 11, 2022 (NCT05322460).

### Participants and Settings

Convenience sampling was conducted during the month of June 2024 among women belonging to the Navarra Breast Cancer Association (Saray), a nonprofit organization supporting individuals with breast cancer in Navarra, Spain. The study invitation was disseminated by Saray through its email newsletter and social media platforms (eg, Facebook and Instagram) and during in-person support group meetings. Women interested in participating were invited to contact the research team via email or telephone, with contact details provided in the invitation. Once contact was established, the research team shared detailed study information, confirmed eligibility, and scheduled the informed consent process.

The inclusion criteria were as follows: (1) being a woman, (2) being aged >18 years, (3) having been diagnosed with breast cancer, (4) having completed active cancer treatments (ie, surgery, chemotherapy, immunotherapy, or radiotherapy) in a period of more than 5 years, (5) being free of disease at the time of data collection, (6) having the ability to use the internet, and (7) having a smartphone. The exclusion criteria were as follows: (1) having a diagnosis of cancer other than breast cancer, (2) having a recurrence of cancer or metastasis for which a new treatment was needed, and (3) being actively treated for recurrence or new cancer.

### CUMACA-M Intervention

This app was developed within the context of the Spanish project titled *CUMACA-M Project: Personalized digital intervention for long-term breast cancer survivors* with the aim of improving the QOL and self-efficacy of long-term breast cancer survivors [23].

**Figure 1.**
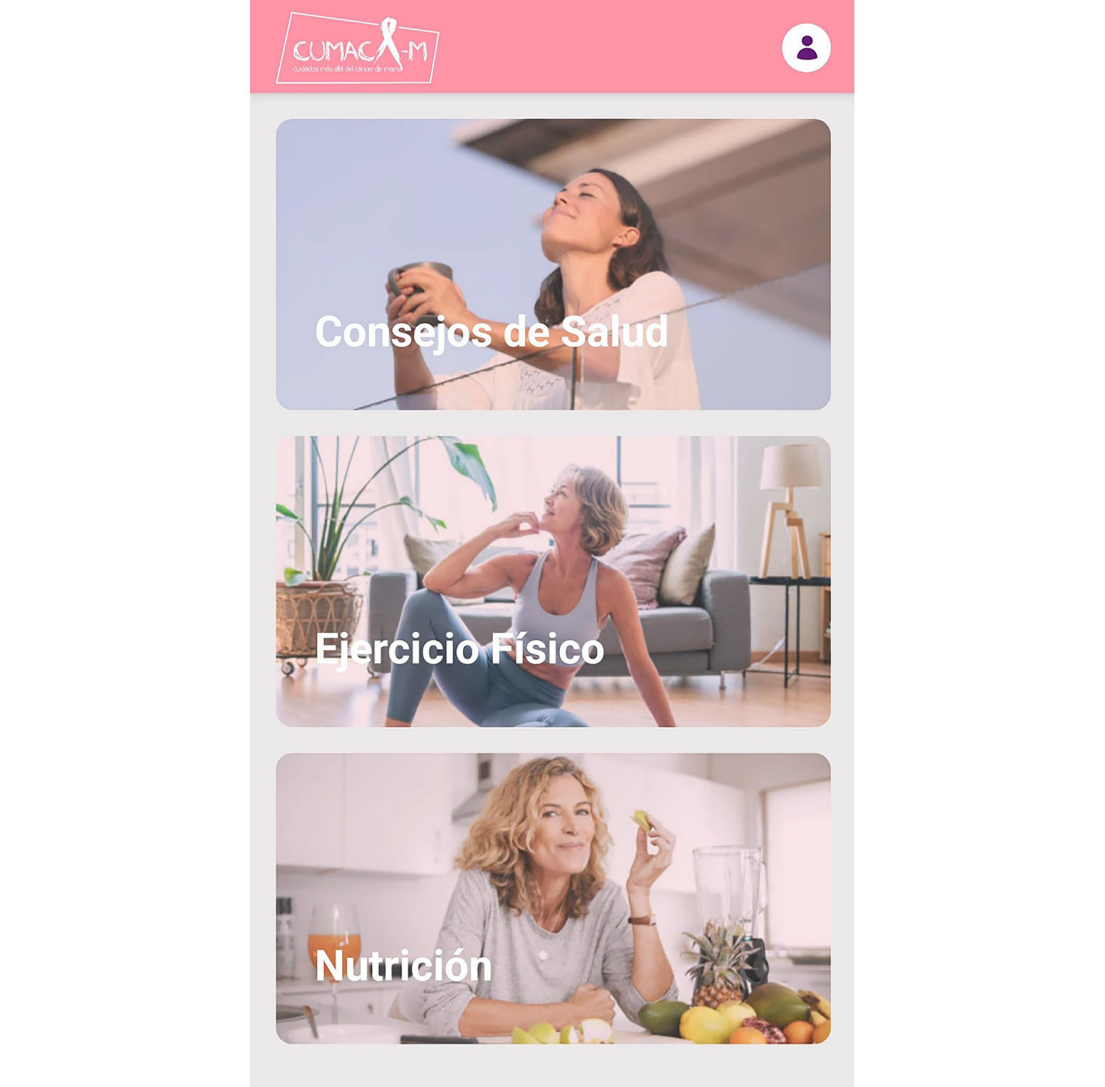
Home screen of the *CUMACA-M* mobile app.

A user-centered design was proposed with a simple and easy-to-navigate interface that required prior registration via email. Artificial intelligence algorithms were incorporated, so the functionalities of the app—including the main modules—could be customized (Table 1); Figure 1). In addition, the app included a notification system with motivational messages.

**Table 1.** Description of the main modules of the CUMACA-M app.

Modules	Functionality
Consejos de salud	Health recommendations: This addressed 22 key categories for long-term breast cancer survivors, including anxiety, wellness, cardiotoxicity, exercise, body image, menopause, nutrition, and sexual health, among others. It provides expert-validated health tips available in text, video, podcast, and infographic.
Ejercicio físico	Physical exercise: A personalized training program was presented. The exercises to be performed, and the duration and the repetitions were explained, accompanied by different videos that demonstrated the execution of the different exercises. The app seamlessly integrated with wearable devices for continuous data collection. This module was programmed based on the main international guidelines for physical exercise, physical activity, and health [27,28].
Nutrición	Nutrition: Personalized daily menus were planned. This module was programmed following international recommendations [29,30].

### Variables and Measurements

The dependent variables analyzed were as follows:

QOL was assessed using the Quality of Life–Cancer Survivors (QOL-CS) scale, originally developed by Ferrell et al [31]. For this study, the validated Spanish version of the instrument was used. The QOL-CS consists of 41 items encompassing four domains: physical well-being (8 items), psychological well-being (18 items), social well-being (8 items), and spiritual well-being (7 items). Each item is rated on a 10-point Likert scale, where 1 indicates extremely poor QOL and 10 represents excellent QOL. The scale has demonstrated high internal consistency, with a global Cronbach α of .93. The α coefficients for the subscales were .77 for physical well-being, .89 for psychological well-being, .81 for social well-being, and .71 for spiritual well-being [31].

Self-efficacy in the management of cancer sequelae was evaluated with the Spanish version of the Self-Efficacy to Manage Chronic Disease Scale (SEMCD-S). This scale consists of 4 items that measure the confidence of individuals in their ability to handle various aspects related to chronic diseases [32]. Each item is scored on a 10-point Likert scale, where 1 indicates “not at all sure” and 10 “totally sure.” The SEMCD-S has high internal consistency, with Cronbach α coefficients ranging from .88 to .95, indicating excellent reliability [32,33].

Moreover, the participants’ sociodemographic data (eg, age, sex, marital status, educational level, employment status) and clinical data (eg, stage of breast cancer at diagnosis, type of treatment(s) received, time elapsed since the end of active cancer treatment, recurrences, and comorbidities) were collected at the beginning of the intervention, using an ad hoc questionnaire.

The usability of the mobile app was evaluated at 3 months using the System Usability Scale (SUS) [34], the most widely used instrument for measuring the perceived usability of digital products and services [35]. It consists of 10 items on a Likert scale, ranging from 1 (“totally disagree”) to 5 (“totally agree”) and has shown high reliability and validity in various studies [36].

The total score ranges from 0 to 100, where higher values indicate better usability. A score of 68 points is considered an average user experience, whereas 78.9 points or more represents excellent usability and 51.7 points or less indicates a poor experience [37]. The Spanish version has shown good content validity (0.92) and easy understanding (face validity of 0.94), with a Cronbach α of .812, reflecting adequate internal consistency [38]. The participants also answered 3 open questions about their experience with the app, their perception of use, and possible improvements.

Feasibility was defined as the proportion of participants who were successfully recruited and who completed both the baseline and follow-up assessments (ie, the QOL-CS and SEMCD-S questionnaires) [39,40]. Acceptability was assessed through usability (SUS) and participants’ responses to 3 open-ended questions about their experience.

### Sample Size

The sample size was estimated following the rules established by Whitehead et al [41] for calculating the sample size of pilot studies on the basis of the minimum desirable effect of 0.075 (standardized difference in QOL), with a standard deviation of 0.18 predicted for the randomized clinical trial. Thus, considering that the SD of this effect (in terms of the QOL variable) was of small intensity (0.1≤*d*<0.3) and that the desired power was 80%, the number of subjects to be recruited was 20.

### Data Procedure

Before the CUMACA-M mobile app was used, it was presented, and its use was reported in an initial session. In this session, the participants signed an informed consent form, downloaded the app, registered (with the username and password), and provided an instruction manual. All individuals who attended this session agreed to participate and signed the informed consent form; no refusals occurred at this stage.

Through the app itself, the data were collected through 2 electronic questionnaires in SurveyMonkey. The first collected sociodemographic and clinical information, whereas the second evaluated QOL (QOL-CS) and self-efficacy in disease management (SEMCD-S). The questionnaires were completed in 2 phases, that is, a preintervention phase (baseline) and a postintervention phase, which were carried out at 3 months. For follow-up, a WhatsApp (Meta Platforms, Inc., Menlo Park, CA, USA) distribution list was created, through which an electronic questionnaire was sent at the end of this period. This included the evaluation of QOL-CS, SEMCD-S, and SUS, in addition to open questions about the experience of using the app: “What are your experiences using the app during the study?,” “How did you perceive the use of the app?,” and “Do you have any suggestions to improve the app?”

### Data Analysis

The data were analyzed using descriptive statistical analysis for quantitative variables (mean and SD) and for qualitative variables (frequencies and percentages). To analyze the changes in the variables QOL and self-efficacy in cancer management before and after using the app, a Student *t* test was used for related samples. All the analyses used a level of statistical significance of *P*<.05. The data were analyzed with SPSS (version 27.0; SPSS Inc, Chicago, IL, USA).

In addition, a content analysis was carried out for the open questions about the experience of the participants with the use of the app. All responses were transcribed verbatim into a spreadsheet. An expert in qualitative methods read the texts to gain an overall understanding and identified meaningful units of text, which were coded and organized into three categories: experiences with the app, perceptions of its utility, and suggestions for improvement. The research team then reviewed these categories and their content, reaching consensus on their relevance and accuracy in representing participants’ perspectives. Finally, representative quotations were selected to illustrate each category.

### Ethical Considerations

This research project complied with the ethical principles established by the Declaration of Helsinki. This study was approved by the Ethics, Animal Experimentation, and Biosafety Committee of the Public University of Navarre (PI-2021/18). Written informed consent was obtained from each participant before the study began. Participants were informed about the objectives of the research, the procedures involved, their right to withdraw at any time without consequences, and the measures taken to ensure confidentiality and data protection.

All data collected were anonymized before the analysis to protect participants’ privacy and confidentiality. No personally identifiable information was retained, and data were stored securely in password-protected files accessible only to the research team.

Participants did not receive monetary compensation for their participation. However, they were provided with detailed information about the study and its potential benefits, and their voluntary involvement was appreciated.

## Results

Twenty-three women, with a mean age of 52.8 (SD 6.1) years and a range between 42 and 61 years, participated. None of the participants withdrew from the study. Most of the participants were married (16/23, 70%), had university studies (15/23, 65%), and used mobile apps regularly (22/23, 96%; Table 2). With respect to healthy habits, no participants reported tobacco use, whereas 17 (74%) participants used alcohol occasionally. With regard to physical activity, 15 (65%) participants performed between 150 and 300 minutes of moderate physical activity a week, 2 (9%) completed 75 to 150 minutes of intense physical activity, and 5 (22%) performed at least 3 days a week of muscle strengthening and balance exercises (Table 2).

**Table 2.** Demographic characteristics and health behaviors of the participants.

Characteristic	Total (N=23)
Age	
Mean (SD)	52.8 (6.1)
Median (range)	53 (42‐61)
Marital status, n (%)	
Married	16 (70)
Divorced	3 (13)
Single	3 (13)
Widowed	1 (4)
Education level, n (%)	
Bachelor or vocational training	6 (26)
Primary	1 (4)
Secondary	1 (4)
University	15 (6)
Using mobile apps regularly**,** n (%)	
No	1 (4)
Yes	22 (96)
Tobacco consumption, n (%)	
No	23 (100)
Alcohol consumption, n (%)	
No	6 (26)
Yes, occasionally	17 (74)
Physical activity, n (%)	
150‐300 minutes of moderate physical activity	15 (65)
75‐150 minutes of intense physical activity	2 (9)
At least 3 days per week of muscle strengthening and balance	5 (22)

Survival time, defined as the period from the end of the primary treatments, had a mean of 7.7 (SD 2.7) years. Of 23 participants, most (21/23, 91%) had a survival time between 5 and 10 years, whereas 2 (9%) exceeded 10 years (Table 3). Regarding the stage of cancer at diagnosis, 6 (26%) women were diagnosed with stage 1 disease, 5 (22%) were diagnosed with stage 2 disease, 3 (13%) were diagnosed with stage 3 disease, and 9 (39%) did not know what stage their disease was in at the time of diagnosis. Lumpectomy was the most frequent surgical procedure, performed in 9 (39%) participants, and 14 (61%) required the removal of more than 1 node. The primary treatments were diverse, with the combination of chemotherapy and radiotherapy being the most common (10/23 44%). A total of 20 (87%) patients received hormonal therapy. In addition, 21 (91%) did not experience cancer relapse.

**Table 3.** Clinical characteristics of the participants.

Characteristic	Total (N=23)
Years since diagnosis, mean (SD)	9.2 (4.1)
Years since completion of primary treatment	
Mean (SD)	7.7 (2.7)
5‐10 years, n (%)	21 (91)
>10 years, n (%)	2 (9)
Stage of cancer, n (%)	
I	6 (26)
II	5 (22)
III	3 (13)
DK^a^	9 (39)
Surgery type, n (%)	
Lumpectomy	9 (39)
Partial mastectomy	7 (30)
Total mastectomy	7 (30)
Removal of lymph nodes, n (%)	
None	1 (4)
1 node	8 (35)
More than 1 node	14 (61)
Type of treatment, n (%)	
None	1 (4)
Chemotherapy	1 (4)
Radiotherapy	5 (22)
Brachytherapy	1 (4)
Chemotherapy+radiotherapy	10 (44)
Chemotherapy+radiotherapy+brachytherapy	1 (4)
Chemotherapy+radiotherapy+immunotherapy	3 (13)
Radiotherapy+immunotherapy	1 (4)
Hormonal therapy, n (%)	
No	3 (13)
Yes	20 (87)
Cancer recurrence, n (%)	
No	21 (91)
1	1 (4)
2	1 (4)

a DK: Don’t know

The classification of the SUS scores is presented in Table 4. The mean score was 80.8 (SD 15.2), corresponding to grade A. Most of the participants (14/23, 61%) reached a score between 84.1 and 100, classified as A+, and 2 (9%) received the lowest score (F).

**Table 4.** SUS^a^ scores according to Lewis and Sauro [37].

Grade	SUS score range	Values, n (%)
A+	84.1‐100	14 (61)
A	80.8‐84.0	1 (4)
A−	78.9‐80.7	2 (9)
B+	77.2‐78.8	0 (0)
B	74.1‐77.1	0 (0)
B−	72.6‐74.0	0 (0)
C+	71.1‐72.5	1 (4)
C	65.0‐71.0	2 (9)
C−	62.7‐64.9	0 (0)
D	51.7‐62.6	1 (4)
F	0.0‐51.6	2 (9)

a SUS: System Usability Scale.

Regarding the experience of use during the study, the most common responses described the app as “easy” (n=7), “simple” (n=5), and “intuitive” (n=7). However, some participants highlighted aspects for improvement, such as that the app “helps, but it is not very specific” (n=1), that “it is sometimes confusing” (n=1), or that “it is not excessively complicated but presents difficulties in managing physical exercise” (n=1). Regarding the perception of use, the majority highlighted the ease of use of the app, although some users indicated that certain aspects could be optimized to improve the experience. Finally, in relation to suggestions to improve the app, the most frequent answer was “no” (n=8), including those that indicated “I do not know” (n=2). However, some participants proposed improvements in the nutrition and exercise modules. For the nutrition module, they suggested incorporating more recipes, including seasonal foods, offering simpler recipes, and using more common ingredients. In the exercise module, they recommended expanding the content and offering more personalization. In addition, 1 of the participants proposed the possibility of face-to-face care from professionals.

Table 5 presents the pre- (baseline) and post- (3 months later) intervention scores obtained on the QOL-CS scales. No statistically significant differences were found in the global score or in most of the subscales of the QOL-CS. A reduction in the spiritual well-being score was observed after the intervention (*P*=.05); however, this finding should be interpreted with caution, given the limited sample size and exploratory nature of the study. For the SEMCD-S scale, no significant changes were observed in any of the items or in the total score (Table 6).

**Table 5.** QOL-CS^a^ instrument scores (domains/subscales).

Domains/subscales	Pre, mean (SD)	Post, mean (SD)	*P* value ^b^
Physical well-being	6.7554 (1.26046)	6.4946 (1.22618)	.23
Psychological well-being	5.1715 (1.33637)	5.1787 (1.43636)	.96
Social well-being	6.5815 (1.78679)	6.2880 (1.88800)	.10
Spiritual well-being	5.3478 (1.13129)	4.9317 (1.22956)	.05
Total score	5.9641 (1.07612)	5.7233 (1.20420)	.07

a QOL-CS: Quality of Life-Cancer Survivors.

b Parametric tests were used: *t* test for paired samples.

**Table 6.** SEMCD-S^a^ instrument scores.

Item	Pre, mean (SD)	Post, mean (SD)	*P* value ^b^
1	7.04 (2.011)	6.48 (1.806)	.20
2	6.30 (2.754)	6.17 (2.329)	.80
3	6.87 (2.138)	6.43 (1.854)	.39
4	6.04 (2.788)	5.96 (1.965)	.86
Total score	6.5652 (1.89979)	6.2609 (1.82077)	.40

a SEMCD-S: Self-Efficacy to Manage Chronic Disease Scale.

b Parametric tests were used: *t* test for paired samples.

## Discussion

### Principal Findings

This pilot study evaluated the feasibility and acceptability of the CUMACA-M mobile app, a digital health intervention incorporating artificial intelligence algorithms and designed to improve QOL and self-efficacy in the management of sequelae among long-term breast cancer survivors. Our findings suggest that the intervention may be feasible, as evidenced by full recruitment and a 100% completion rate. All participants completed the evaluations using the QOL-CS, SEMCD-S, and SUS scales, which reflect high adherence and commitment to the study. The initial training session may have contributed to these positive outcomes by reducing potential technical barriers and facilitating user engagement, thereby minimizing dropout and ensuring completion of follow-up assessments. This level of participation is especially relevant because the permanent survival stage presents significant challenges for recruiting participants. In many cases, people who have completed their cancer treatment tend to withdraw from studies related to the disease either because they want to focus on their recovery or because they prefer not to emotionally relive their experience with cancer [42].

In addition, the intervention was well accepted by the participants, as evidenced by the results obtained on the SUS scale and the qualitative responses on the experience of using the app. The high score in the SUS (mean 80.8, SD 15.2) indicates excellent usability of the system, which suggests that the app is well designed and functional for this population. These results are consistent with those of previous studies that evaluated the feasibility of mobile apps targeting breast cancer survivors. For example, Bergqvist et al [43] reported an average score of 82.5 on the SUS (standard deviation not reported), with positive perceptions of its ease of use and added value in daily life. Monteiro-Guerra et al [44] reported an average value of 95.0 (SD 6.3), with a high assessment in terms of its ease of use and impact on promoting physical activity. However, unlike these studies, our intervention is the first designed specifically for breast cancer survivors who are in the permanent or long survival stage (disease-free and more than 5 years from the completion of primary cancer treatment).

On the other hand, the participants identified various strengths of the platform, as well as areas for improvement. In particular, the health tips module received no optimization comments, which can be attributed to the close collaboration with users during its development, a strategy recommended in previous studies [43-45], which probably contributed to the high perceived usability. However, the nutrition and physical exercise modules, which had less participation from users in their design phase, were the subject of observations and suggestions for their improvement. These findings underscore the importance of actively involving the study target population in the process of developing digital interventions to maximize their acceptance and effectiveness.

Regarding the results obtained for QOL and self-efficacy in the management of sequelae, which were evaluated using the QOL-CS and SEMCD-S scales, no statistically significant differences were detected between the pre- (baseline) and post- (3 months later) measurements. These findings suggest that, during the period analyzed, the perceived QOL and self-efficacy of the participants in managing their chronic condition remained stable. These findings can be explained by several factors. First, the stability of the measurements could indicate that the intervention helped maintain the levels of QOL and self-efficacy, avoiding the possible deterioration associated with the time and progression of the sequelae. In addition, 3 months may not be sufficient to observe significant changes in these variables because improvements in the perception of QOL and in self-efficacy usually require longer periods of intervention and follow-up. On the other hand, individual factors, such as previous health status, degree of adaptation to life after cancer, or variability in the use of the intervention, could have influenced the results, attenuating the expected effects.

These results are consistent with those of the scientific literature, which has reported heterogeneous results concerning the impact of digital interventions on the QOL of breast cancer survivors [22,46-49]. While some studies have documented significant improvements in certain domains of QOL [22,46], others have not identified statistically significant differences [22,50], and some research has even reported negative effects associated with intervention [49]. The systematic review of Pimentel-Parra et al [22] reflects this variability, reporting positive effects of digital health in aspects such as physical activity, fatigue, sleep disorders, anxiety, and depression, but without significant differences in other dimensions such as vitality, physical role, or self-efficacy for exercise.

Ultimately, the findings of this study highlight the complexity of evaluating the impact of digital interventions on the QOL of long-term breast cancer survivors. In addition, they highlight the need for a longer period to detect clinically relevant changes in QOL and self-efficacy for the management of sequelae. Future studies with a longer design and larger sample sizes will allow a more precise evaluation of the impact of these interventions on QOL and self-efficacy for the management of sequelae in long-term survivors of breast cancer. In addition, it is essential to consider additional variables, such as adherence to the intervention, the degree of participation of the users, and psychosocial factors that may influence the results. Likewise, the incorporation of mixed methodologies, combining quantitative and qualitative analyses, could provide a deeper understanding of the experience of the participants and the mechanisms through which these interventions generate changes in their well-being.

### Strengths and Limitations of the Study

The strengths and limitations of this study are acknowledged, providing a comprehensive view of its contributions and considerations for future research. First, although the literature suggests that usability studies typically require a minimum of 15 to 20 participants to obtain reliable results [51,52], this study included 23 long-term breast cancer survivors, slightly exceeding the initially estimated sample size by 15%. This greater participation strengthens the validity of the findings and highlights the viability of recruiting and maintaining the commitment of this population in digital health interventions. In this sense, the absence of participant dropout may indicate a favorable level of commitment and acceptance of the intervention over the 3-month period, which could suggest the potential feasibility of implementing similar tools in clinical and community settings for the follow-up of long-term breast cancer survivors. However, generalizability remains limited due to the sample size and specific study context, so caution should be exercised in interpreting these outcomes, and further investigation is needed.

The inclusion of participants with varied ages, health conditions, and levels of technological experience could enhance the relevance of the findings for a broader population of long-term breast cancer survivors, although this diversity does not fully compensate for the limited scope of the sample. The findings may provide a preliminary foundation for future improvements in the design and development of digital support strategies tailored to long-term breast cancer survivorship, with possible apps in follow-up and rehabilitation contexts. Finally, the combination of the SUS with open-ended qualitative questions allowed for a more nuanced understanding of the user experience, highlighting both perceived strengths and areas for further improvement.

However, one of the main limitations of this study is its pilot nature, as it had a relatively small sample size and a short evaluation period. Although the 100% completion rate is an encouraging finding that supports the feasibility of the intervention, it is possible that this result was influenced, in part, by the shortness of follow-up. In fact, previous studies have shown that adherence and completion rates tend to decrease with longer-term interventions due to factors such as user fatigue, loss of interest, or the presence of long-term logistical barriers [50,53,54]. In addition, no objective monitoring of the activity of the users was carried out within the app, which prevents the precise evaluation of the patterns of use and the actual adherence to the intervention. In this sense, future research with longer follow-up periods will allow a more realistic assessment of sustained adherence and the long-term impact of the intervention.

In addition, although long-term breast cancer survivors may experience common side effects and late effects, this study did not include a systematic assessment of whether participants themselves had these experiences. As such, it remains unclear to what extent the sample reflects the broader survivorship population in terms of symptom burden. This limits the ability to draw conclusions about the intervention’s applicability or effectiveness for individuals facing persistent treatment-related sequelae. Future studies should incorporate measures to assess these factors and examine their influence on user engagement and intervention outcomes.

### Conclusions

This pilot study suggested that the CUMACA-M mobile app may be feasible and acceptable for long-term breast cancer survivors, as indicated by a high completion rate and positive usability feedback. While these preliminary findings are encouraging, the small sample size and the specific context in which the study was conducted limit the generalizability of the results. Further research with larger and more diverse samples is needed to confirm these findings and to better understand the role of co-creation in the development of digital health interventions for this population.

Future studies with larger samples, prolonged follow-ups, and objective metrics of use could more precisely evaluate the impact of CUMACA-M on the QOL and self-efficacy of breast cancer survivors.

## Supplementary material

10.2196/76719 Checklist 1 TREND Statement checklist.

## Abbreviations

CUMACA-M: Care Beyond Breast Cancer, in Spanish
ICT: information and communication technology
QOL: quality of life
QOL-CS: Quality of Life-Cancer Survivors
SEMCD-S: Self-Efficacy to Manage Chronic Disease Scale
SUS: System Usability Scale
